# Inhibitions of Several Antineoplastic Drugs on Serum Sialic Acid Levels in Mice Bearing Tumors

**DOI:** 10.3797/scipharm.1209-18

**Published:** 2012-11-14

**Authors:** Da-Yong Lu, Jing Xu, Ting-Ren Lu, Hong-Ying Wu, Bin Xu

**Affiliations:** 1 School of Life Sciences, Shanghai University, Shanghai 200444, PR China.; 2 College of Science, Shanghai University, Shanghai 200444, PR China.; 3 Shanghai Institute of Materia Medica, Chinese Academy of Sciences, Shanghai 201203, PR China.

**Keywords:** Sialic acids, Antineoplastic drugs, Neoplasm metastasis, Cancer therapy, Probimane, Cisplatin, Nitrogen mustard, Lycobetaine

## Abstract

Six murine tumors, including ascetic tumors HepA, EC, P388 leukemia, S180 and solid tumor S180, and Lewis lung carcinoma, were employed in this work. The free sialic acid concentrations in both blood and ascites were measured in tumor-bearing mice. The results showed that the content of sialic acids in blood was increased in tumor growth and certain tumor types. Higher sialic acid content was observed in ascites than that present in blood. The influence of antineoplastic agents (vincristine, thiotepa, adriamycin, probimane, cisplatin, oxalysine, cortisone, nitrogen mustard, lycobetaine, Ara-C, harringtonine, and cyclophosphamide) on the content of sialic acids in mice blood bearing solid tumors of either S180 or Lewis lung carcinoma was observed. Different inhibitions of antineoplastic drugs on both tumor growth and serum sialic acid levels in mice bearing tumors were found. Among these antineoplastic drugs, probimane, cisplatin, nitrogen mustard, and lycobetaine were able to decrease the serum sialic acid levels in mice bearing tumors. Since these four antineoplastic drugs are all DNA chelating agents, it was proposed that the inhibition of tumor sialic acids by these drugs might be through the DNA template via two ways. Since we have found no effect of antineoplastic drugs on serum sialic acid levels in normal mice, this suggests that the inhibition of antineoplastic drugs on sialic acids is by tumor involvement.

## Introduction

Cancer metastasis is responsible for 90% of all cancer deaths. However, current therapeutic approaches to this pathogenesis is unsatisfactory [1, 2]. One of the reasons is the lack of therapeutic targets specifically related to neoplasm metastasis. Cancer metastasis is a long-term cascade in humans and leads to a series of biological, biochemical, and pathophysiological consequences [3–5]. We previously argued that more therapeutic targets specifically related to neoplasm metastasis should be studied [6], especially the sialic acid pathways [7–8]. In this paper, the inhibitions of serum sialic acid levels in mice bearing tumors by antineoplastic drugs are studied.

Sialic acids (neuraminic acids) are a special series of 9-carbon backbone acidic carbohydrates, and are typically found at the outermost part of sugar chains attached to cell macromolecules. They play many important roles in a series of pathophysiological processes, including microbe binding that leads to infections, regulation of the immune response, the progression and spread of human malignancies, and in certain aspects of human evolution [9, 10]. The earliest work tackling the phenomenon of a positive relationship between sialic acids and tumors can be traced back to Kimura et al from 1958 [11, 12]. Their discovery is that tumor cells might excrete and contain more sialyl glycoproteins or glycolipids. These characteristics later have been found to be linked with highly metastatic tumor types [13]. Many researchers have shown that patients with tumors of high levels of sialyl antigens appear to be linked with a poor prognosis [14, 15], which is one of the most clinically conspicuous pathologic features of sialic acids in tumors. Now, many sialyltransferases or sialidases have been found to express relatively higher or lower levels in tumors than in normal tissues [16–19]. Since more than 50 different types of sialic acid monosaccharides have ever been discovered [20], they can be linked with other normal monosaccharides (heptose or hexose and so on) to form tremendously diversified 2–6 sugar component antigens (sugar chains)—sialic acids are often at the farthest end of antigens and glycoproteins. Among these antigens, some of them are very tumorigenic and widely occur among different tumors, such as sialyl Lewis X and A, which are known to positively correlate with colon and non-small cell lung cancer and core α6-fucosylation with liver and pancreatic cancer [14, 15]. With all of this evidence, we think sialic acids in neoplasms might be a novel therapeutic target.

## Materials and methods

Antineoplastic drugs were purchased from local pharmaceutical companies or prepared by the Division of Medicinal Chemistry, Shanghai Institute of Materia Medica, Chinese Academy of Sciences.

5-Acetyl-neuraminic acids (Neu5Ac, NANA)—the most prevalent of sialic acid analogues—and bovine serum albumin (BSA) were purchased from Sigma Chemical Company, USA. Laboratory animals were purchased from the Shanghai Center of Laboratory Animal Breeding, Chinese Academy of Sciences, and experiments were conducted in compliance with the Guidelines for the Care and Use of Research Animals, NIH, established by Washington University’s Animal Studies Committee.

Murine tumors (Hep A, EC, S180, P388, Lewis lung carcinoma) were serially transplanted in different strains of mice by the Division of Anticancer Drug Pharmacology, Shanghai Institute of Materia Medica, Chinese Academy of Sciences.

### The determinations of sialic acid levels in mice bearing tumors

**Fig. 1:**
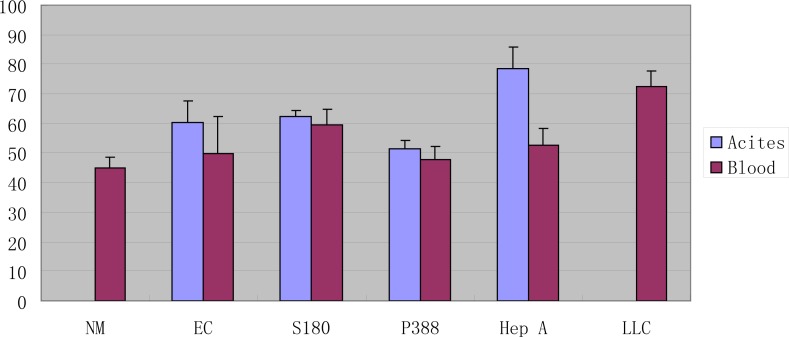
Serum or ascites sialic acid levels of mice bearing different tumors; NM normal mice, LLC Lewis lung carcinoma

Ehrlich carcinoma, Hep A, and S180 sarcoma were intraperitoneally or subcutaneously transplanted into normal albino mice. P388 leukemia was intraperitoneally transplanted into the DBA strain of mice and Lewis lung carcinoma (3LL) was subcutaneously transplanted into the C57/BL strain of mice. After 10 days, the tumor ascites and blood were drawn from mice and added to normal saline (1/9) and centrifuged (2000 g) for 10 min. Total sialic acids [21] and protein [22] content in the supernatant of saline were determined. The ratio of sialic acids (mg) to proteins (g) was plotted in Figure 1.

### Therapeutic study of antineoplastic drugs on serum sialic acid levels in mice bearing solid tumors

The solid forms of sarcoma S180 or 3LL were subcutaneously transplanted into the albino or C57/BL strain of mice. These mice were given (ip×7) antineoplastic drugs from day 2 of tumor inoculations. On day 11, the blood of mice was drawn and added with normal saline. The total contents of sialic acids and proteins in the supernatant of saline were determined (see above). Then, mice were humanely sacrificed. The primary tumors were weighed and the percentages of tumor inhibition by drugs were calculated.

### The effects of antineoplastic drugs on serum sialic acid levels in normal mice

Normal mice were given antineoplastic drugs at the same dosages as previously injected into mice bearing tumors. On day 11, the mice were humanely sacrificed and serum sialic acid levels were determined.

### Statistical analysis

The statistical analysis of data is by Student’s T-test

## Results

### The sialic acid contents in ascites and blood of mice bearing tumors

Figure 1 shows that there is an increase in serum sialic acid levels in mice bearing different tumors. Among them, the increases in serum sialic acid levels in mice bearing solid tumors are higher than that in mice bearing ascetic tumors. The sialic acid levels of ascites are relatively higher than those in the blood of mice bearing tumors.

### The inhibitions of some antineoplastic drugs [21, 22] on serum sialic acid levels in mice bearing tumors

Table 1 and Table 2 show that probimane 10–30 mg/Kg ip×7 and cisplatin (DDP) 4mg/Kg ip×7 can inhibit the growths of S180 and 3LL. In the meantime, they inhibit serum sialic acid levels (P<0.05) in mice bearing S180 and 3LL.

Nitrogen mustard (NM) 1mg/Kg ip×7 and lycobetaine 10 mg/Kg ip×7 inhibited serum sialic acid levels in mice bearing S180 (p<0.05) and also inhibited serum sialic acids levels in mice bearing LLC. Harringtonine 0.2 mg/Kg ip×7 had increased serum sialic acid levels in mice bearing both S180 and 3LL. Adriamycin 2 mg/Kg ip×7 and cortisone 20 mg/Kg ip×7 could not inhibit the growth of 3LL, but could increase serum sialic acid levels in mice bearing 3LL. Thiotepa, Ara-C, Oxalysine, vinblastine, and cyclophosphamide have shown to inhibit tumor growth but had no effect on serum sialic acid levels in mice bearing either S180 or 3LL (Table 1 and 2).

### No effect of antineoplastic drugs on serum sialic acid levels in normal mice

In this experiment, no effect of antineoplastic drugs on serum sialic acid levels in normal mice was found.

## Discussion

In this experiment, we found there were higher sialic acid levels in ascites than those in serum. It suggests that increased levels of sialic acids might be produced by tumor tissue and then diluted in ascites and further in the blood. In this method, we have determined the sialic acid contents of both sialyl-lipids and sialyl-protein—total sialic acids content. It is an important parameter for us to understand the overall sialic acid status of tumor-bearing mice.

In this experiment, the dosages of antineoplastic drugs we used were relatively small, which were supposed to be antimetastatic dosages. In these dosages, we could determine the drug inhibitions on serum sialic acids. Here, we found that probimane, cisplatin, nitrogen mustard, and lycobetaine could inhibit serum sialic acid levels in mice bearing tumors. Of these four antineoplastic drugs, probimane is an antimetastatic agent [23, 24]. Cisplatin and nitrogen mustard are well-known DNA chelating agents and lycobetaine is also a DNA binding agent [25]. So, it is proposed that inhibitions of tumor sialic acids by these drugs might be through a DNA template via two ways.

1. DNA→RNA→proteins (CMP-sialic acid synthase, sialyltransferases, and sialidase)
2. DNA→unknown mechanisms→sialyl-conjugators

In the previous work, other researchers have reported that some antimetastatic agents had inhibited sialic acid levels in mice bearing the highly metastatic tumor cell line B16-F10 [26, 27]. It seems there is a solid relationship between tumor sialic acid inhibition and tumor metastatic inhibition. In this work, we further suggest that this relationship is correlated with tumor DNA synthesis inhibition.

It might be through the unknown “glycobiology central paradigm” to fulfill this mechanism, and needs our further work. Here, we found that cortisone could increase serum sialic acid levels in mice bearing tumors. We all know that cortisone is an inhibitor of the body’s immune system. Since sialic acids participate in a wide range of physiological and pathologic processes, including human immune reactions [9, 10], this work provides new insight into this matter. As harringtonine is a protein synthesis inhibitor, the promotion of serum sialic acid/protein levels by harringtonine might be through the inhibition of protein synthesis, and may further increase the ratio of sialic acid to protein.

Hence, we found no effect of antineoplastic drugs on serum sialic acid levels in normal mice. It suggests that the inhibition of antineoplastic drugs on sialic acids is by tumor involvement.

## Figures and Tables

**Tab. 1.** Effects of several antineoplastic drugs on serum sialic acid levels in mice bearing S180

**Compound**	**Dosage mg/Kg mg/Kg**	**Mean body weight Initial/end**	**Tumor inhibition %**	**Serum sialic acids μg/protein/mg (X±SD)**
Control	–	21.6/31.6	–	60.0±9.7
Ara-C	100	21.5/27.8	14.3	58.7±7.5
Harringtonine	0.2	21.7/31.4	4.3	77.5±5.0^**^
Control	–	18.3/26.8	–	62.5±17.5
Cortisone	20	18.2/26.2	23.3	67.5±18.0
NM	1	18.4/23.4	33.1	41.3±12.3^**^
Control	–	25.2/26.4	–	60.0±8.7
Probimane	30	24.6/27.0	80.3	53.5±5.0^*^
CTX	20	25.2/27.5	1.9	57.5±10.7
Lycobetaine	10	24.6/28.7	−51.0	52.3±5.7^*^
Oxalysine	20	25.0/26.7	−12.5	55.0±18.7
DDP	4	24.6/27.0	20.2	38.7±7.3^**^

Schedule ip×5;

* P<0.05;

** P< 0.01. There were 5 mice in each group.

**Tab. 2.** Effects of several antineoplastic drugs on serum sialic acid levels of mice bearing Lewis lung carcinoma

**Compound**	**Dosage mg/Kg mg/Kg**	**Mean body weight Initial/end**	**Tumor inhibition %**	**Serum sialic acids μg/protein/mg (X±SD)**
Control	–	19.5/22.9	–	52.5±6.2
Vinblastine	0.2	19.6/22.7	−17	51.2±12.0
Thiotepa	2	19.8/22.8	4	56.7±12.2
Control	–	18.3/25.1	–	75.2±7.5
Adriamycin	2	18.1/23.2	–	86.3±14.7^*^
Control	–	20.2/33.7	–	77.6±5.7
Probimane	10	20.3/27.2	17.5	60.0±4.7^**^
DDP	4	20.0/23.4	29.9	52.9±6.2^**^
Control	–	21.2/32.6	–	54.4±18.0
Oxalysine	20	21.2/32.2	21.5	52.4±12.0
Cortisone	20	21.2/26.3	−7.6	70.0±18.0^*^
Control	–	22.8/28.2	–	53.5±9.5
Ara-C	100	22.5/27.8	50	57.8±7.5
Harringtonine	0.2	22.7/28.5	−6.5	62.2±17.5^*^
NM	1	22.9/21.2	51.9	49.5±7.5
Control	–	18.7/20.0	–	63.7±9.7
Lycobetaine	20	18.5/19.2	30.8	56.8±12.0

Schedule ip×5;

* P<0.05;

** P<0.01. There were 5 mice in each group.
